# Is Placebo Acupuncture What It Is Intended to Be?

**DOI:** 10.1093/ecam/nep049

**Published:** 2011-06-18

**Authors:** Thomas Lundeberg, Irene Lund, Audrey Sing, Jan Näslund

**Affiliations:** ^1^Foundation for Acupuncture and Alternative Biological Treatment Methods, Sabbatsbergs Hospital, Sweden; ^2^Department of Physiology and Pharmacology, Karolinska Institutet, Sweden; ^3^Foundation for Acupuncture and Alternative Biological Treatment Methods, Sabbatsbergs Hospital, Sweden; ^4^Department of Physiology and Pharmacology, Karolinska Institutet, Stockholm, Sweden

## Abstract

Randomized, placebo-controlled clinical trials are recommended for evaluation of a treatment's efficacy with the goal of separating the specific effects (verum) from the non-specific ones (placebo). In order to be able to carry out placebo-controlled acupuncture trials, minimal/sham acupuncture procedures and a sham acupuncture needle has been used with the intention of being inert. However, clinical and experimental results suggest that sham/minimal acupuncture is not inert since it is reported that both verum acupuncture and sham/minimal acupuncture induce a significant alleviation of pain. This alleviation is as pronounced as the alleviation obtained with standard treatment and more obvious than the one obtained with placebo medication or by the use of waiting list controls. These results also suggest that sham acupuncture needles evoke a physiological response. In healthy individuals sham acupuncture results in activation of limbic structures, whereas a deactivation is seen in patients with pain, i.e. results from healthy individuals do not reflect what is seen in clinical conditions. Also, depending on the etiology of pain (or any under clinical condition under investigation), the response to sham acupuncture is varying. The acupuncture ritual may also be seen as an emotional focused therapy allowing for psychological re-orientation. Sham needling in such context may be as powerful as verum acupuncture. We recommend that the evaluated effects of acupuncture could be compared with those of standard treatment, also taking the individual response into consideration, before its use or non-use is established.

## 1. Introduction

During the last decade, a large number of randomized controlled trials (RCT) have been published comparing manual acupuncture or electro-acupuncture with different modes of intended placebo controlled procedures in the treatment of perceived pain. The placebo control procedures most commonly used include minimal or superficial acupuncture (needling of the skin), sham acupuncture (deep or superficial needling of non-acupuncture points) and the use of placebo acupuncture needles (a blunt tip of a needle touches the skin without penetrating it) [1–3]. The intention of these RCTs is to reduce the presence of bias of the results by comparing the size of the interventional specific effects, by means of assumed, specific mechanisms, with the non-specific effects of an inert (placebo) comparator applied in a placebo-controlled procedure. This trial design is considered the gold standard in evaluation of all types of intervention and its result forms the basis for further determination of the effectiveness of acupuncture.

Recent studies performed in Germany have evaluated the effects of *minimal acupuncture* (superficial needling outside disease specific acupuncture points according to traditional Chinese medicine—TCM) and *acupuncture* (needles inserted into classical acupuncture points according to TCM, and manually stimulated until the dull radiation sensation of deqi was evoked) in patients with migraine, low back pain and knee osteoarthritis pain. In general, there was no marked difference in efficacy between minimal acupuncture and acupuncture [4]. Thus, most of the effects of acupuncture have by some been attributed to unspecific placebo responses [5]. However, another possibility is that minimal acupuncture produces specific effect and is consequently not inert. If so, the present research trial design (placebo acupuncture versus acupuncture) is not valid. Furthermore, instead of reducing bias there is a risk of introducing bias against the findings of the tested treatment. This suggestion is supported by the findings of the German RCTs, Tables 1, 2, and 3, showing that placebo acupuncture (here specified as minimal acupuncture) is as effective as standard treatment and more effective than placebo medication in reducing migraine [6–10]. Interestingly, in low back pain [11–13] and knee osteoarthritis pain [14–16], acupuncture had a better effect as compared to placebo acupuncture. The divergent results in the different pain conditions could be interpreted as that the effects of the sensory stimulation produced by placebo acupuncture and acupuncture is dependent on the etiology of pain. Also, the minimal acupuncture technique is only minimal in a TCM perspective. In a psycho-physiological perspective minimal acupuncture is anything but inert.

## 2. Etiology of Pain

Pain is often considered to be a homogeneous sensory entity, mediated by a specialized high threshold sensory system, which extends from the periphery through the spinal cord, brain stem and thalamus to the cerebral cortex. However, multiple mechanisms have been detected in the nervous system responsible for pain of different etiologies [17]. When pain is transformed from an acute (an alarm signal) to a chronic state (a sustained challenge) part of the brain areas is re-organized. Furthermore, this re-organization continuous also in the chronic state, having an impact on the cortex producing unique pattern [18]. Apart from the pain influence on the cortical function there is a continuous reorganization affecting the supraspinal brain areas responsible for descending modulation of pain. Acute and chronic pain also has different impact on learning and memory [18], which should be considered when assessing the efficacy of acupuncture.

## 3. Placebo

A positive placebo response is seen in a varying degree in patients with pain, Parkinson's disease and depression. The placebo response has been reported being more pronounced with invasive procedures or advanced disease [19–21] and, neuroimaging studies have provided a major contribution to our understanding of the mechanisms of the placebo effects. Expectation of symptom improvement has long been believed to play a critical role in the placebo effect and is suggested to be driven by frontal cortical areas, particularly the dorsolateral prefrontal, orbitofrontal and anterior cingulate cortices. The ventral striatum is involved in the expectation of rewarding stimuli and, together with the prefrontal cortex, has also been shown to play an important role in the placebo-induced expectation of therapeutic benefit. Also, positron emission tomography studies have shown that the placebo effect is related to the activation of the limbic circuitry. The observation that placebo administration induces the release of dopamine in the ventral striatum of patients with Parkinson's disease suggests a link between the placebo effect and reward mechanisms. In addition to Parkinson's disease, the placebo-reward model may also apply to other disorders. However, the relative contribution of the different neurotransmitters and neuropeptides that are known to be involved in modulating the activity of the limbic system may be disease-specific. Thus, while the placebo-induced clinical benefit observed in Parkinson's disease would mostly reflect the release of dopamine in the dorsal striatum, the activation of opioid and serotonin pathways could be particularly implicated in mediating placebo responses encountered in pain and depression, respectively [19–21].

## 4. The Physiological Complexity of Acupuncture Effects

Acupuncture is a non-uniform treatment where for instance all, Modern, physiological and Traditional, approaches is used. Besides, depending on how it is carried out, different results may be obtained [22]. Responses to the induced stimulation in different endogenous systems have been shown to play key roles in acupuncture analgesia such as the endogenous opioid system and the descending serotoninergic inhibitory pathway [23]. However, as described above, the basic function of these systems have been shown to be altered as a consequence of the pain that also is depending on the etiology of the pain, explaining why different modes of acupuncture, including low and high frequency electro-acupuncture, may have different effects (i.e., being the most effective modality). Except endogenous opioids and serotonin, the cholecystokinin octapeptide (CCK-8) has been shown to be important in the effects of acupuncture including development of tolerance. The individual differences of acupuncture analgesia are also associated with inherited genetic factors and the density of CCK receptors. Furthermore, depending on the characteristics of the pain, such as spontaneous, persistent or stimulus-evoked, and its related default mode of the brain, different modalities of acupuncture including the stimuli evoked by placebo acupuncture may have different effects in the patients [24]. In general, patients with musculoskeletal pain of inflammatory or ischemic origin showed the largest probability to obtain pain alleviation whereas in patients with neuropathic pain this was less likely and even so less in patients with persistent (idiopathic) pain [24].

## 5. Effects of Placebo Acupuncture in Pain and Other Conditions

Several studies have reported that the placebo acupuncture procedure is adequate from an expectation perspective, that is, acupuncture naïve patients cannot reveal which is real and which is control. During placebo acupuncture procedures, stimulation of the cutaneous touch receptors and/or skin nociceptors may be activated [25, 26]. The activity set up in these receptors is conveyed into the brain and result in the modulation of the activity in the brain areas included in the pain neuromatrix such as the limbic structures [27–30]. In healthy subjects, the acupuncture results in increased activity in the limbic structures whereas in patients with pain a deactivation of the same structures is reported [31–33]. This would suggest that trials using healthy subjects is of great interest but have limited clinical relevance. Also, pains with different etiology may be associated with different characteristics, like spontaneous or stimulus evoked pain. These symptoms may be differently susceptible to the influence of acupuncture. In pain patients with persistent pain the stimulus response to most modalities of sensory stimulations is augmented (central sensitization), whereby for example light stimulation of the skin is perceived as being very strong and in some cases even painful [34]. Also, in patients with persistent pain the receptive fields of central nociceptive neurons are expanded, resulting in a larger topographic distribution in the “higher” levels of central nervous system of the pain [21].

Except for modulating the activity in the hypothalamus and the limbic structures, the activity set up in afferent nerves during placebo acupuncture modulates the reward system resulting in a sensation of wellbeing [35–39]. It can also be assumed that the clinical context of the acupuncture treatment may serve as behavioral conditioning suggesting that the (repeated) needling ritual per se contributes to the therapeutic effects of acupuncture [40]. In a psychological perspective acupuncture may be viewed as an emotion focused therapy were placebo acupuncture or acupuncture results in alterations in the functional connectivity making the patient more susceptible to an emotional re-orientation [41]. One factor that has been suggested to explain a major part of the effects of sham acupuncture in irritable bowel syndrome (IBS) is the interaction between the therapist and the patient [42]. However, this interaction is in itself also context dependent making general conclusions about sham acupunctures efficacy in patient-therapist relations in other conditions invalid [43] since patients treated with acupuncture due to allergic rhinitis physician characteristics played a minor role in the effectiveness of acupuncture treatment [44].

In IBS patients treated with acupuncture or placebo acupuncture the quality of life improved in both groups with no group differences. Furthermore, a more pronounced salivary cortisol decreased was seen in the acupuncture group as was the decrease of the heart rate response during orthostatic stress indicating an increased parasympathetic tone in the acupuncture group. Also, improvement of pain was positively associated with increased parasympathetic tone in the acupuncture group but not in the placebo group. Thus, different mechanisms seem to be involved in placebo acupuncture and acupuncture driven improvements [45]. That cortisol release is decreased after acupuncture is further supported by a study on patients with chronic low back pain, subjected to different modalities of acupuncture (traditional Chinese acupuncture, sham acupuncture, electro-acupuncture and electro-acupuncture at non-acupuncture points) [46]. A significant decrease in plasma cortisol concentration was measured after all interventions suggesting that the decrease was a centrally controlled, hypothalamic medicated response. However, the changes in cortisol concentrations in plasma are dependent on the condition treated. In patients suffering from “environmental illness” and treated with acupuncture or placebo acupuncture, cortisol concentrations increased. In that study both groups improved significantly during and after treatment without any group differences. The changes seen in biological variables were gradual with a continuous increase in serum cortisol and a decrease in neuropeptide Y [47].

Recently, the use of acupuncture during *in vitro* fertilization (IVF) treatments has attracted interest as it has been suggested to improve outcome, that is, pregnancy rate. In a randomized double blind study, aimed to compare acupuncture with placebo acupuncture in patients undergoing IVF treatment, 370 patients were randomly allocated to either acupuncture or placebo acupuncture before embryo transfer. Interestingly the overall pregnancy rate was significantly higher in the placebo acupuncture group than that in the acupuncture group resulting in the suggestion that “Placebo acupuncture may not be inert” [48].

## 6. Conclusion

Acupuncture as used currently is not a standardized treatment and likely several acupuncture techniques owns the potential of inducing clinical treatment effects depending on the condition treated. Experimental and clinical studies have shown that the acupuncture placebo procedures applied are not inert, (from a psycho-physiological perspective) and should therefore not be interpreted as placebo-controls in RCTs for the test of efficacy, that is, the present research trial design (placebo acupuncture versus acupuncture) may be questioned. Instead of reducing bias, it introduces a bias against the findings of the acupuncture treatment. The introduction of the placebo needle was a brilliant idea, however, it is up to the user to determine what its use may reflect and how its effect should be interpreted in an evidenced based medicine perspective [49, 50]. The minimal acupuncture technique may be regarded a valid control of TCM medicated effects but not for effects of acupuncture as a modality of sensory stimulation.

## Figures and Tables

**Table 1:** Results, treatment of migraine.

Intervention: Author, year (ref.); number of included subjects (*n*)	Proportion patients (in %) reporting reduced frequency days with migraine after treatment [5–9]
Acupuncture	
Linde, 2005 [6]; *n* = 302	51
Streng, 2006 [7]; *n* = 114	61
Diener, 2006 [8]; *n* = 960	47
Minimal acupuncture	
Linde, 2005 [6]; *n* = 302	53
Diener, 2006 [8]; *n* = 960	39
Medication	
Diener, 2002 [9]; *n* = 808	46 (Flunarizin 5 mg)
	53 (Flunarizin 10 mg)
	48 (Propanolol)
van der Key, 2002 [10]; *n* = 2013	46 (Propanolol)
Streng, 2006 [7]; *n* = 114	49 (Metropolol)
Diener, 2006 [8]; *n* = 960	40 (Standard medication)
Placebo tablet	
van der Key, 2002 [10]; *n* = 2013	24
Waiting list	
Linde, 2005 [6]; *n* = 302	15

**Table 2:** Results, treatment of low back pain.

Intervention: Author, year (ref.); number of included subjects (*n*)	Proportion patients (in%) reporting decreased low back pain [10–12] and increased function [11, 12] after treatment
Acupuncture	
Brinkhaus, 2006 [11]; *n* = 298	54
Haake, 2007 [12]; *n* = 1162	48
Witt, 2006 [13]; *n* = 3093	53
Minimal acupuncture	
Brinkhaus, 2006 [11]; *n* = 298	39
Haake, 2007 [12]; *n* = 1162	44
Standard treatment	
Haake, 2007 [12]; *n* = 1162	27
Routine care	
Witt, 2006 [13]; *n* = 3093	27
Waiting list	
Brinkhaus, 2006 [11]; *n* = 298	15

**Table 3:** Results, treatment of knee osteoarthritis pain.

Intervention: Author, year (ref.); number of included subjects (*n*)	Proportion patients (in%) reporting decreased knee osteoarthritis pain and increased function [13–15]
Acupuncture	
Witt, 2005 [14]; *n* = 294	52
Scharf, 2006 [15]; *n* = 1007	53
Witt, 2006 [16]; *n* = 712	35
Minimal acupuncture	
Witt, 2005 [14]; *n* = 294	28
Scharf, 2006 [15]; *n* = 1007	51
Standard care	
Scharf, 2006 [15]; *n* = 1007	29
Routine care	
Witt, 2006 [16]; *n* = 712	7
Waiting list	
Witt, 2005 [14]; *n* = 294	3
